# Week-by-Week Predictive Value of External Load Ratios on Injury Risk in Professional Soccer: A Logistic Regression and ROC Curve Analysis Approach

**DOI:** 10.3390/medicina61111954

**Published:** 2025-10-30

**Authors:** Andreas Fousekis, Konstantinos Fousekis, Georgios Fousekis, Gregory Bizas, Sotiris Vino, Gerasimos Paraskevopoulos, Georgios Gounelas, Panagiotis Konomaras, Yiannis Michailidis, Andreas Stafylidis, Athanasios Mandroukas, Nikolaos Koutlianos, Iosif Gavriilidis, Thomas Metaxas

**Affiliations:** 1 Laboratory of Evaluation of Human Biological Performance, Department of Physical Education and Sports Science, Aristotle University of Thessaloniki, 57001 Thessaloniki, Greece; ioannimd@phed.auth.gr (Y.M.); astafylidis@phed.auth.gr (A.S.); amandrou@phed.auth.gr (A.M.); tommet@phed.auth.gr (T.M.); 2 Therapeutic Exercise and Sports Rehabilitation Laboratory, Physiotherapy Department, University of Patras, 26504 Patras, Greece; konfousekis@gmail.com (K.F.); gio.fousekis@gmail.com (G.F.); 3 School of Physical Education and Sport Science, National and Kapodistrian University of Athens, 17237 Athens, Greece; gregorybizas@hotmail.com (G.B.); spetimvino1987@gmail.com (S.V.); 4 Department of Physical Education and Sport Science, Democritus University of Thrace, 69100 Komotini, Greece; geparask@phyed.duth.gr (G.P.); geogoun17@hotmail.com (G.G.); panos_konomaras@hotmail.com (P.K.); 5 Sports Medicine Laboratory, School of Physical Education and Sport Science, Aristotle University of Thessaloniki, 57001 Thessaloniki, Greece; koutlian@phed.auth.gr; 6 Interbalkan Medical Center, 55535 Thessaloniki, Greece; igavriilid@yahoo.gr

**Keywords:** injury prevention, professional soccer, football, ACWR, injury prediction

## Abstract

*Background and Objectives*: This study aimed to assess the week-by-week predictive value of Acute:Chronic Workload Ratios (ACWRs) for non-contact injury risk in professional soccer players. *Materials and Methods*: A cohort of 40 elite players was monitored using GPS over two competitive seasons. Binomial logistic regression and ROC curve analyses were performed on ACWR metrics—including total distance, moderate-to high-speed running, sprinting, acceleration, and deceleration—during the four weeks prior to injury (W4 to W1). *p*-values were further adjusted for multiple comparisons using the false discovery rate (FDR) correction (q < 0.05). *Results*: Significant predictive models emerged mainly for ACWR metrics related to moderate-speed running (15–20 km/h), sprinting (>25 km/h), and acceleration/deceleration. The ACWR for 15–20 km/h (DSR15–20) demonstrated the highest predictive accuracy, particularly in Week 3 (AUC = 0.811, *p* = 0.004). Sprinting (DSR>25) was also significantly associated with injury occurrence across Weeks 1–4 (AUC = 0.709–0.755, *p* = 0.011–0.024). Acceleration (ACC) and deceleration (DEC) metrics showed significant associations prior to correction—ACC in Weeks 3–4 (AUC = 0.737–0.755, *p* = 0.020–0.026) and DEC in Weeks 3–4 (AUC = 0.720–0.741, *p* = 0.029–0.043)—but these associations did not retain significance following FDR adjustment (q = 0.052–0.086). In contrast, total distance (ACWR TD) and high-speed running (20–25 km/h) were weaker predictors, reaching only marginal or nonsignificant levels (e.g., Week 3, AUC = 0.675, *p* = 0.054). After FDR correction, only DSR15–20 and DSR>25 remained statistically significant (q < 0.05), confirming them as robust predictors of non-contact injury risk. Multivariable models adjusted for age and playing position confirmed these findings, with DSR15–20 and DSR>25 retaining their predictive value independent of confounding factors. Injury risk thresholds were established through Estimated Marginal Means (EMMs), defining the “Sweet Spot” and “Danger Zone” for each metric, whereas the “Low Load” zone was treated as exploratory. *Conclusions*: This weekly ACWR monitoring approach enables practical injury risk profiling, helping training staff optimize load management and minimize non-contact injury risk in elite soccer.

## 1. Introduction

Previous studies have explored the association between Acute:Chronic Workload Ratio (ACWR) and injury occurrence, often suggesting a “sweet spot” range for workload metrics such as total distance and high-speed running. However, these studies frequently combined data from multiple sports, limiting sport-specific applicability. Furthermore, predictive models quantifying week-by-week injury risk remain scarce. A focused investigation within elite professional soccer is needed to provide actionable insights tailored to the demands of the sport. This study provides a novel week-by-week stratified analysis of ACWR in the four weeks preceding non-contact injuries. Using binomial logistic regression and ROC curve analysis, it identifies specific time windows and workload metrics that are most predictive of injury risk. The findings offer actionable insights for technical staff to monitor and adjust training loads in real time. The methodology enhances injury forecasting and supports evidence-based load management strategies in elite soccer environments.

Soccer stands as the world’s most widely played and followed sport [1]. In recent years, however, the physical demands of training and competition have risen dramatically, resulting in a heightened risk of injury—particularly to the lower limbs. This has made injury prevention a critical priority for both players and coaching staff [2]. Many of these injuries involve soft tissue damage, which not only affects short-term performance but may also signal an underlying predisposition to re-injury [3]. Elevated training loads often exacerbate muscular and joint asymmetries, reducing stability, mobility, and neuromuscular control—sometimes triggering compensatory load shifts to the opposite limb [4].

According to Inklaar’s classification [5], injury risk factors in soccer are divided into endogenous (internal) and exogenous (external/environmental) categories. Among exogenous factors, key contributors include physical contact with opponents [2], inappropriate training loads [6,7], insufficient protective gear [3], and variations in playing surfaces [8]. Of these, training load variation plays a particularly important role in both performance development and injury prevention. As a result, daily monitoring of training load has become an integral aspect of professional teams’ planning, supporting progressive overload while helping to prevent injury-inducing stress.

Crucial metrics for assessing external load include total distance, high-speed running, sprints, accelerations, and decelerations. The accurate weekly calculation of these variables allows technical staff to compare current workloads with previous weeks, helping to optimize performance while reducing injury risk. The overarching goal—aligned with our study—is to implement effective injury prevention strategies through continuous tracking of an athlete’s workload over time.

There is debate in existing research regarding training intensity: some studies advocate for moderate exposure to high-intensity work to prevent injuries [9], while others suggest frequent high-intensity drills are necessary to prepare for match-day extremes [10]. In both cases, precision in weekly load calculation is essential to avoid fatigue-related risks that can impact subsequent performances [11].

This study aims to offer evidence-based guidelines for weekly training load management, particularly identifying ACWR (Acute: Chronic Workload Ratio) thresholds that should not be exceeded, as well as the external load indicators most closely linked to injury. Accordingly, the study seeks to answer the following primary research question: Which weekly ACWR thresholds and external load variables most accurately predict the likelihood of non-contact injury in professional soccer players? A secondary research question is also examined: Do variations in ACWR values across consecutive weeks alter the probability of injury occurrence, thereby identifying critical periods of heightened risk? By analyzing weekly load trends and comparing current data with that from previous weeks, the goal is to understand individual limits and avoid excessive stress that can lead to non-contact injuries. Although GPS data is widely collected, there is limited research synthesizing this data into a coherent injury-risk model over multiple weeks. Our research addresses this gap, exploring external load patterns over the four weeks preceding an injury, with the aim of identifying the most injury-critical periods.

In prior studies, conflicting interpretations have emerged regarding which of the four weeks before injury should be evaluated and which load parameters carry the most weight. Our approach clarifies this by focusing on key metrics such as total distance, running speeds across multiple zones (15–20, 20–25, 25–30 km/h), accelerations > 2.5 m/s^2^, and decelerations > 2.5 m/s^2^, assessed across all four weeks (W-4 to W-1). The speed zones were chosen due to the known thresholds present in high-level soccer [12], which corresponded to gradual increases in both metabolic demand and mechanical strain on the musculoskeletal system. Finally, compared to focusing only on total accumulated ACWR values for injury prediction, the present study presents a weekly stratified approach that allows us to provide more precise insight into when within the four-day modeled risk window the imbalance between acute and chronic load becomes most predictive. This methodological improvement may serve to clarify the temporal sensitivity of ACWR as an indicator of injury risk. This comprehensive two-year dataset offers unique insight into the significance of each week and the risk thresholds, particularly ACWR values approaching or exceeding 1.3, which may signal increased risk of injury, which is confirmed by a systematic review that notes a trend for ratios of 0.80–1.30 [13].

Supporting this investigation, previous research on 369 athletes found that 206 players (55.8%) sustained a total of 538 lower-limb injuries over two seasons [14]. Many of these injuries are linked to neuromuscular deficits such as reduced strength and anaerobic power. Furthermore, re-injury rates are high: in one study of 57 soccer players, 64% of those with previous lower-limb injuries still exhibited muscle imbalances upon return [15].

The theoretical basis for our analysis is the ACWR, a method that evaluates weekly (acute) workload against the average of the previous four weeks (chronic) [16,17,18,19,20,21,22]. Originally developed to measure the interplay of fitness and fatigue, ACWR is now more commonly used as a predictor of injury risk. Deviations from the optimal ACWR window (generally between 0.80 and 1.30) are increasingly seen as indicators of non-contact injury risk [19].

Another important consideration is player position, which significantly affects workload demands. Comparisons are most meaningful when made among players with similar tactical roles [23], as GPS data typically reflects consistent patterns within positional groups. Although some studies [24] have assessed this, few have gone in-depth into how load differs by position or its relationship to injury risk. Notably, midfielders appear most injury-prone (43.6%), followed by defenders (30.0%) and forwards (17.9%) [25].

GPS tracking plays a vital role in quantifying external load and has been shown to reduce injury rates when used to monitor week-to-week load changes [24]. Tiernan [26] found that an increase in acute workload of ≥9% from one week to the next is associated with higher injury risk, reinforcing the need for gradual training progression.

While research on the load–injury relationship is still emerging, this study seeks to fill significant gaps by providing practical, detailed guidance for injury prevention. Unlike prior research, we assess all four weeks leading up to an injury, offering new insights into which week or metric poses the greatest risk. This approach allows for the real-time comparison of training microcycles, helping technical staff evaluate load fluctuations and manage them proactively.

Specifically, the present study sought to investigate the association between ACWR and non-contact injury risk in professional soccer players, using a weekly stratified analysis. It was hypothesized that higher ACWR, specifically for high-speed running (HSR) at 20–25 km·h^−1^ and sprint distance (≥25 km·h^−1^), would be associated with an increased likelihood of injury within 1–3 weeks prior to injury onset compared to more distant periods prior to injury based on previous research [19]. This hypothesis is based on the assumption that external load exerted intensely above an optimal ratio to the connective tissue’s adaptive capacity can cause a cumulative microstructural damage, neuromuscular fatigue, and incomplete recovery (which) can cause short-term deterioration of tissue tolerance, accompanied by suboptimal coordination to prevent injury.

Based on the existing research data, there is a gap in the literature regarding the recording mainly of the last weeks before an injury. There are also conflicting parameters regarding the external load that will cause a non-contact injury, but also regarding the time period that should be evaluated. It is observed that the risk of non-contact injury was 5–6 times higher for low-intensity accelerations and distances in one particular study, with ACWR defined as >2.0 (RR = 5.4–6.6), when all chronic loads were included, with ACWR > 2.0 being significantly associated [18]. In contrast, in another study [27], in 1-week accumulated loads and acute/chronic workload ratios (ACWRs), no differences in accumulated weekly loads and ACWR calculations were observed between muscle injuries (*p* > 0.05).

## 2. Materials and Methods

### 2.1. Experimental Design

This study utilized a prospective longitudinal observational cohort design and players’ training load and match exposure were quantified, with time-loss injuries recorded over one competitive season. While previous studies have analyzed load correlations two and four weeks prior to injury [23], the present study expands on this by examining all four weeks (W-4 to W-1) leading up to the injury event. The research spans two competitive seasons (2021–2022 and 2023–2024) and includes 40 professional soccer players. During the competition microcycles, twenty non-contact soccer players were injured with the specific injuries totaling 4.5/1000 h. The determination of the minimum required sample size was conducted using G*Power (version 3.1.9.7) for a z-test logistic regression model. The analysis was designed to estimate the sample necessary to detect large effects under realistic model conditions derived from the present dataset. Parameters were specified as follows: odds ratio (OR) = 6.0, α = 0.05 (two-tailed), R^2^ other X = 0.10 (representing moderate shared variance with additional predictors), Pr (Y = 1|X = 1) = 0.50, and X-distribution = binomial (π = 0.50). The computation indicated that a total sample of N = 41 participants would provide approximately 60% statistical power to detect an odds ratio of this magnitude. The final dataset comprised 40 professional soccer players, thus closely matching the theoretical requirement. Given that the observed effects in the logistic regression analyses were large (OR = 4–8; Nagelkerke R^2^ = 0.20–0.40), the achieved power was considered acceptable for exploratory analysis within a homogeneous elite athletic cohort.

External load data were captured using 10 Hz GPS units (Vector S7, Catapult Sports Ltd., Melbourne, Australia), following the reliability protocols established by Clavel [28].

In total, 300 training sessions across 50 training microcycles and 60 official matches were analyzed. During certain weeks, 24 microcycles included an additional midweek match. All training and match sessions were conducted on natural grass outdoor pitches, maintaining environmental consistency throughout the study.

The Checklist statement for planning was established by the Statistical Evaluation of Medical Practices (CHAMP).

### 2.2. Participants

The study sample comprised professional soccer players from Greece’s two major leagues (Super League 1 and Super League 2), observed over two full seasons (The sample concerned soccer players who competed in Super League 1 but had the opportunity to compete in Super League 2 during the same season.). The soccer players were specifically divided into two research groups. Of the 40 football players who participated in the research process, a sample of 20 football players who were injured during the period in which the data were recorded was needed and another sample of 20 football players who did not show an injury.

The injuries were classified as minimal (1–3 days of loss of active participation), mild (4–7 days of loss), moderate (1–4 weeks of loss) and severe (4+ weeks of loss) [29]. Overall, the injuries recorded in the study were mild and moderate, according to the previous classification. In the muscle groups of the lower extremities that were injured during the study, it is observed that the hamstring area holds the largest percentage, followed by the quadriceps with the adductors, while at the end there are the gastrocnemius. Also in the characterization regarding the size of an injury, moderate injuries account for the largest percentage, followed by mild and minimal injuries and serious injuries. Contact injuries were completely excluded from the analyses or analyzed separately, while the two observed seasons concerned the same team and the same coaching staff.

Injured players’ load data were compared to non-injured counterparts who shared the same positional role, ensuring tactical and physical demand equivalence. For every injury event, the 4 weeks prior to the injury (weeks −4 through −1) were selected as the injury exposure period.

Reference periods for non-injuries were randomly selected from players with at least 4 consecutive weeks without an injury and matched by position and period of season (early, middle or late). Contextual effects (e.g., match congestion and opponent level) were accounted for during matching in an attempt to minimize temporal bias.

More specifically, data was analyzed for 4 central defenders, 10 full-backs, 12 central midfielders, 4 wingers and 10 forwards. A total of 40 players participated (mean age: 20.6 ± 1.6 years; height: 179.7 ± 4.8 cm; weight: 76.8 ± 4.3 kg; body fat: 8.6 ± 1.3%). All participants were thoroughly briefed on the procedures and provided informed consent before the study. Ethical approval was obtained from the Aristotle University of Thessaloniki Ethics Committee (Ref: 96/2021) in accordance with standard research practices in sports and exercise science. Patients and the public were not involved in the design, conduct, reporting, or dissemination plans of this research.

### 2.3. Running Load and Weekly Monitoring

GPS devices were positioned on the upper back of each player, and the same device was used by each athlete throughout the study to ensure data consistency. These devices have been validated for reliability in previous research [30,31] and are widely used in professional soccer contexts [1,31,32,33].

Measured metrics included: Total distance covered, high-speed running (14.4–19.8 km/h), very high-speed running (19.8–25.0 km/h), sprints (>25.0 km/h), accelerations and decelerations (>2.5 m/s^2^). Specific values (>2.5 m·s^−2^) for accelerations and decelerations have been defined and used in similar research [34]. This limit satisfactorily ensures a high-intensity energy.

Weekly training data were collected within a microcycle structure, based on the model by Owen [7]. Each weekly training block was coded according to its relationship to match day (MD): Six-day microcycle: MD+1 (recovery), MD+2 (rest), MD-4, MD-3, MD-2, MD-1, Seven-day microcycle: MD+1 (recovery), MD+2 (rest), MD-5 to MD-1, Eight-day microcycle: MD+1 (recovery), MD+2 (rest), MD-6 to MD-1, Nine-day microcycle: MD+1 (recovery), MD+2 (rest), MD-7 to MD-1.

All athletes were familiar with the structure and procedures. Importantly, only full participation data were included—players had to complete the entire training program and participate in matches. Goalkeepers and players under load restrictions (due to injury prevention or management) were excluded, ensuring the integrity of the dataset.

To determine the initial ACWR value prior to the microcycle in which the soccer player sustained an injury, data from four weeks, three weeks, two weeks, and the week of the injury were used. The chronic workload was first calculated based on the average loads from the fourth, third, second, and first weeks preceding the injury. The acute workload, referring to the load from the injury week itself, was then divided by this chronic workload to compute the ACWR. In other words, the ACWR represents the ratio of the athlete’s workload during the injury week to the average workload over the previous four, three, two, or one week(s). ACWR was calculated using a simple rolling average and was defined as a total of four weeks (W1–W4) with each week, for example, “W3” representing the third week before injury.

The data collection and processing process was applied daily in the study. Only non-contact injuries were included. A doctor always gave the final opinion on an injury after all the necessary medical examinations had been performed. Only non-contact time-loss injuries were involved, which were described as any physical complaint incurred during training or match play not involving direct player-to-player or player-object contact that led to at least 1 missed training session or match. The injury classification was performed based on the consensus-criteria by UEFA and the severity of injuries was defined according to time loss: minimal (1–3 days), mild (4–7 days), moderate (8–28 days), and severe (>28 days). All the diagnoses were made by the medical doctor of the team and confirmed. None of the players suffered from repeat injuries, which were identified as an injury occurring at the same site involving the same type twice or more after full recovery.

### 2.4. Equity, Diversity, and Inclusion (EDI) Statement

This study was designed and conducted in accordance with principles of equity, diversity, and inclusion (EDI). The participant cohort consisted exclusively of male professional soccer players from Greece’s Super League 1 and Super League 2, reflecting the demographic structure and competitive environment of the leagues under investigation. While the inclusion criteria were determined by the scope of the study and the availability of data within these male leagues, all eligible participants were treated equally and without bias in data collection and analysis. The authors acknowledge the importance of sex and gender diversity in sports science research and recommend future investigations to include female athletes and underrepresented groups to enhance the generalizability and inclusivity of findings.

### 2.5. Statistical Analysis

The determination of the minimum required sample size was conducted using G*Power (version 3.1.9.7) for a z-test logistic regression model. The analysis was designed to estimate the sample necessary to detect large effects under realistic model conditions derived from the present dataset. Parameters were specified as follows: odds ratio (OR) = 6.0, α = 0.05 (two-tailed), R^2^ other X = 0.10 (representing moderate shared variance with additional predictors), Pr (Y = 1|X = 1) = 0.50, and X-distribution = binomial (π = 0.50). The computation indicated that a total sample of N = 41 participants would provide approximately 60% statistical power to detect an odds ratio of this magnitude. The final dataset comprised 40 professional soccer players, thus closely matching the theoretical requirement. Given that the observed effects in the logistic regression analyses were large (OR = 4–8; Nagelkerke R^2^ = 0.20–0.40), the achieved power was considered acceptable for exploratory analysis within a homogeneous elite athletic cohort.

A series of binomial logistic regression analyses were performed to evaluate the association between various ACWR indicators and the probability of injury occurrence across a four-week observational period (Weeks 1–4). Separate logistic regression models were estimated for each ACWR variable, including total distance (ACWR TD), moderate-speed running (ACWR DSR15), high-speed running (ACWR DSR20), sprinting (ACWR DSR25), acceleration load (ACWR ACC), and deceleration load (ACWR DEC). Each predictor was modeled independently at each week (e.g., ACWR TD W1 to ACWR TD W4), resulting in a total of 24 models. Model fit was evaluated using Akaike Information Criterion (AIC), McFadden’s pseudo-R^2^ (R^2^McF) and Nagelkerke R^2^ (R^2^N) values. Statistical significance of each model was determined via the likelihood ratio chi-square test, while parameter-level inferences were based on the Wald test, with significance set at *p* < 0.05. Specifically, the model χ^2^ (likelihood ratio test) assessed whether the inclusion of the ACWR predictor significantly improved overall model fit compared with the null model, whereas the Wald test evaluated the statistical significance of the individual regression coefficient (β_1_) within each model. For each predictor, odds ratios (ORs) and 95% confidence intervals (CIs) were reported to quantify the magnitude and direction of associations. To assess predictive performance, each model’s classification table was used to compute accuracy, sensitivity and specificity, using a decision threshold of 0.50. Additionally, Receiver Operating Characteristic (ROC) curve analyses were conducted for each model to estimate the Area Under the Curve (AUC), that reflects the model’s ability to discriminate between injured and non-injured cases. The 95% confidence intervals (CIs) for the AUC values were calculated using the approximation proposed by Hanley and McNeil [35]. Specifically, the standard error (SE) of AUC was computed as:

$${SE}_{AUC}=\sqrt{\frac{AUC(1-AUC)+(N_{1}-1)(Q_{1}-{AUC}^{2})+(N_{2}-1)(Q_{1}-{AUC}^{2})}{N_{1}N_{2}}}$$

where $Q_{1}=\frac{AUC}{2-AUC}$ and $Q_{2}=\frac{2AUC^{2}}{1+AUC}$ with $N_{1}$ and $N_{2}$ representing the number of positive (injury) and negative (non-injury) cases, respectively. The 95% confidence interval was then derived as $AUC\pm 1.96\times SE_{AUC}$. A model was considered to have discriminative ability beyond random guessing if its 95% CI excluded 0.5. Pairwise comparisons of AUC values between models were further conducted using the nonparametric method proposed by DeLong et al. [36], which allows for statistical testing of correlated ROC curves derived from the same sample. In the present analysis, the correlation coefficient between AUCs was set at r = 0.5, reflecting moderate dependency among models evaluated on the same dataset.

For predictors approaching significance, Estimated Marginal Means (EMMs), formerly known as Least Squares Means (LSMeans), were calculated at standardized levels (−1 SD, mean, +1 SD) to visualize the probability of injury associated with varying workload ratios. These EMMs estimate what the mean probability of injury would be at each workload level if all other model parameters were held equal, thereby facilitating more accurate and unbiased comparisons between workload conditions. The area under the ROC curve (AUC) was interpreted according to Hosmer et al. [37], where values between 0.7 and 0.8 indicate acceptable discrimination, 0.8–0.9 excellent discrimination, and values above 0.9 represent outstanding discrimination. Selected models with statistically significant predictors and/or higher predictive utility (AUC > 0.70) were further illustrated using combined EMM plots and ROC curves to aid interpretation. All analyses were conducted using Jamovi (version 2.6). Statistical significance was defined as *p* < 0.05.

To account for the increased risk of Type I error due to multiple hypothesis testing across 24 independent models (6 ACWR indicators × 4 weeks), *p*-values obtained from the Wald tests were further adjusted for multiple comparisons using the Benjamini–Hochberg (BH) procedure [38]. The false discovery rate (FDR) approach was applied within each ACWR variable family (m = 4 weeks per variable), thus controlling for multiple testing over time within each load category rather than across all models simultaneously. Specifically, raw *p*-values were ranked in ascending order (p_(i)_), and adjusted q-values were computed using the formula q_(i)_ = min[(p_(i)_ × m)/rank_(i)_, 1], followed by a step-up monotonic correction ensuring that adjusted *p*-values were non-decreasing with rank (q_(i)_ = min(q_(i)_, q_(i+1)_, …, q_(m)_)). This method provides a less conservative and more powerful alternative to traditional Bonferroni correction while effectively controlling the expected proportion of false positives. After adjustment, only ACWR DSR15–20 and ACWR DSR > 25 remained statistically significant across multiple weeks (q < 0.05), whereas all other predictors, including ACWR TD, DSR20–25, ACC and DEC, did not survive the FDR correction (q > 0.05). This approach ensures that reported significant findings reflect robust associations rather than spurious effects driven by multiple model testing.

The thresholds defining the “Low Load Zone”, “Sweet Spot Zone” and “Danger Zone” were derived from the analysis of EMMs, which were estimated during the application of binomial logistic regressions, for each weekly ACWR variable. For each ACWR variable the software computed the mean, mean −1 SD and mean +1 SD for ACWR and predicted the probability of injury [(P (injury)] at those three values using the logistic regression equation:

$$P\left(injury\right)=\frac{1}{1+e^{-(\beta 0+\beta 1\times ACWR)}}$$

where β0 = intercept, β1 = coefficient of the ACWR variable.

Specifically, for each ACWR predictor, the mean and standard deviation (SD) were computed, and three representative values were selected: the mean, mean −1 SD and mean +1 SD. These three values (mean ±1 SD and mean) were used to generate predicted probabilities of injury at defined points along the workload. The Low Load Zone was defined as the range of ACWR values below the lower threshold (mean –1 SD), where training stimulus was minimal. The Sweet Spot Zone corresponded to the interval between −1 SD and +1 SD, where injury probability remained moderate or controlled. In contrast, the Danger Zone was identified as the range exceeding +1 SD, where injury probability increased, indicating potential overload. Then, these zones were visualized in Excel using line charts overlaid with colored sections to represent their boundaries, in line with the theoretical framework for the Sweet Spot and Danger Zone proposed by Gabbett [19].

## 3. Results

Binomial logistic regression analyses (Table 1) were performed to investigate the predictive value of various ACWR metrics for injury occurrence across a four-week period (W1–W4). Across the two competitive seasons, an average of 0.4 injuries per week was recorded. The most frequently affected muscle group was the hamstrings (35.0%), followed by the quadriceps (25.0%) and adductors (25.0%), while the gastrocnemius accounted for 15.0% of all injuries. Each model included a single ACWR predictor, categorized by type of external load (total distance [TD], distance at 15–20 km/h [DSR15–20], 20–25 km/h [DSR20–25], >25 km/h [DSR > 25], acceleration [ACC] and deceleration metrics [DEC]).

**Figure 1:**
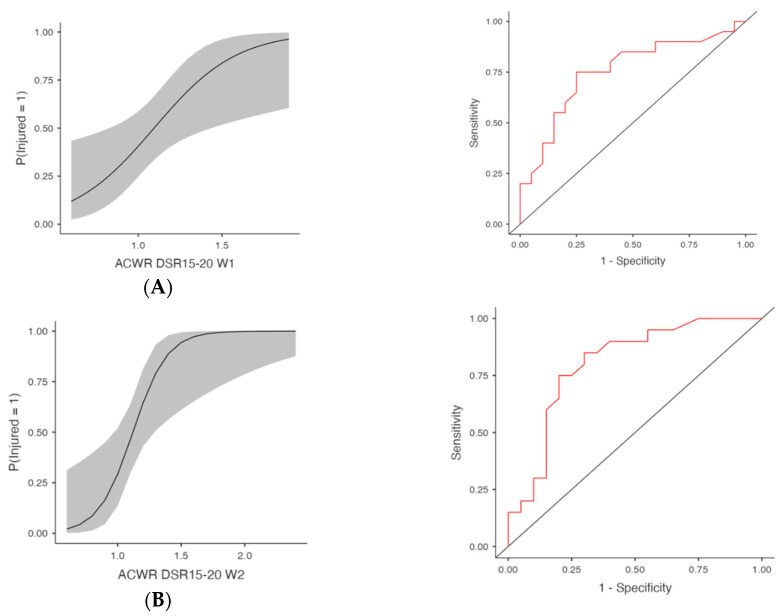

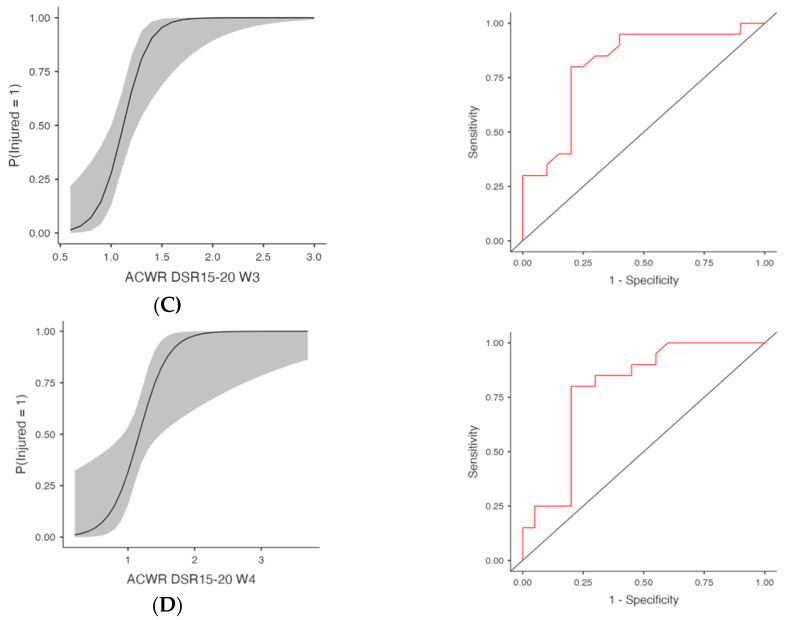
Injury Risk Probability and ROC Curves for Weekly ACWR DSR15-20. Note: (**Left**) panels display the estimated probabilities of injury (Injured = Yes) as a function of acute:chronic workload ratios (ACWRs), with 95% confidence intervals (shaded area). (**Right**) panels show the ROC curves corresponding to each predictor, assessing the discriminative ability of the logistic regression models. The red lines represent the sensitivity–specificity trade-off across probability thresholds, with the area under the curve (AUC) quantifying model performance. (**A**) Week 1 (W1): Probability of injury and ROC curve based on ACWR DSR15-20 values from the first week preceding injury occurrence. (**B**) Week 2 (W2): Probability of injury and ROC curve based on ACWR DSR15-20 values from the second week preceding injury occurrence. (**C**) Week 3 (W3): Probability of injury and ROC curve based on ACWR DSR15-20 values from the third week preceding injury occurrence. (**D**) Week 4 (W4): Probability of injury and ROC curve based on ACWR DSR15-20 values from the fourth week preceding injury occurrence.

**Figure 2:**
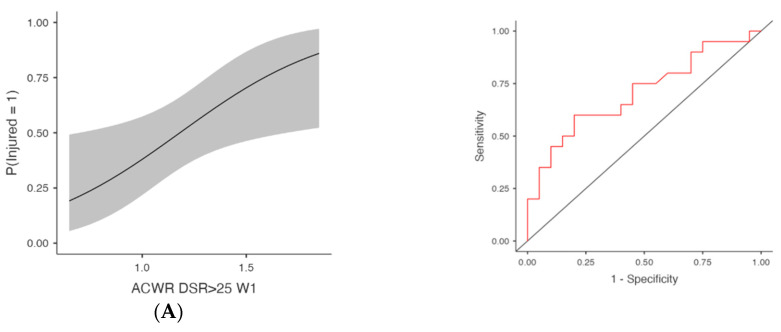

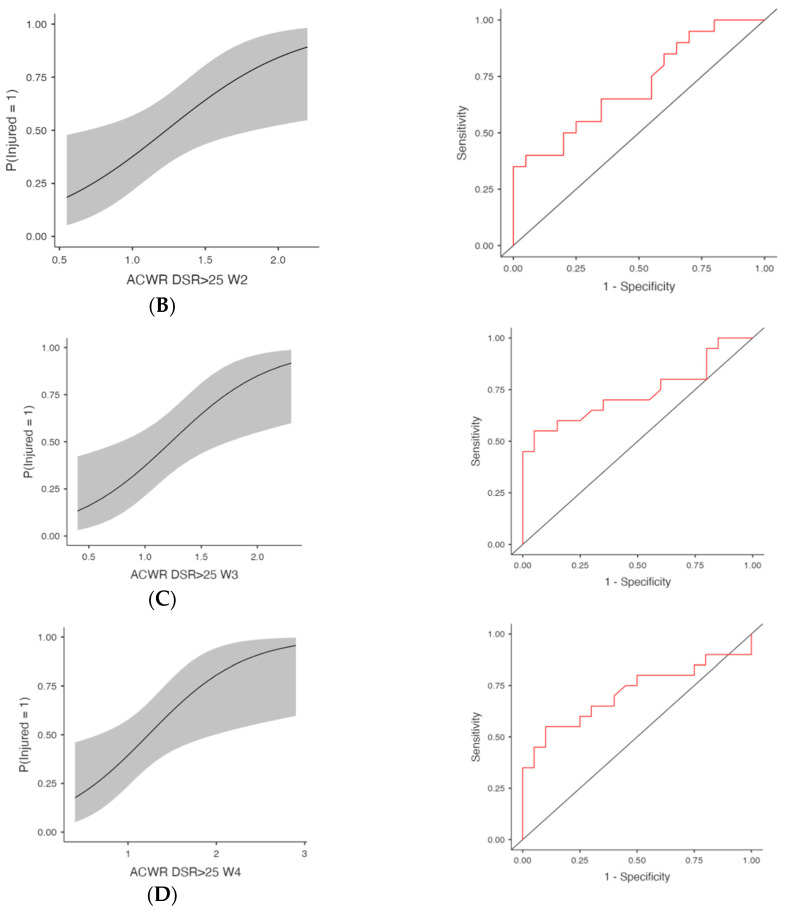
Injury Risk Probability and ROC Curves for Weekly ACWR DSR>25. Note: (**Left**) panels display the estimated probabilities of injury (Injured = Yes) as a function of acute:chronic workload ratios (ACWRs), with 95% confidence intervals (shaded area). (**Right**) panels show the ROC curves corresponding to each predictor, assessing the discriminative ability of the logistic regression models. The red lines represent the sensitivity–specificity trade-off across probability thresholds, with the area under the curve (AUC) quantifying model performance. (**A**) Week 1 (W1): Probability of injury and ROC curve for the ACWR of sprinting distance (>25 km/h) recorded during the first week preceding injury occurrence. (**B**) Week 2 (W2): Probability of injury and ROC curve for the ACWR of sprinting distance (>25 km/h) recorded during the second week preceding injury occurrence. (**C**) Week 3 (W3): Probability of injury and ROC curve for the ACWR of sprinting distance (>25 km/h) recorded during the third week preceding injury occurrence. (**D**) Week 4 (W4): Probability of injury and ROC curve for the ACWR of sprinting distance (>25 km/h) recorded during the fourth week preceding injury occurrence.

**Figure 3:**
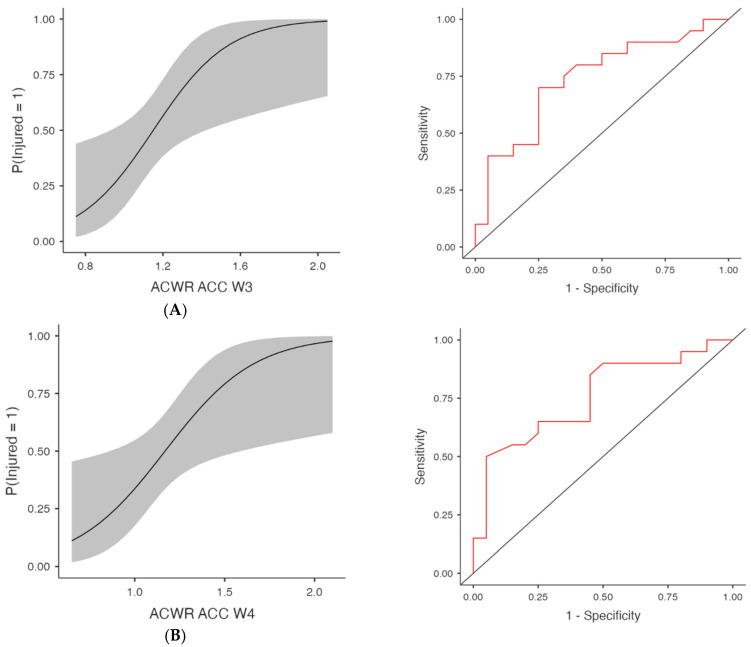
Injury Risk Probability and ROC Curves for Weekly ACWR- Acceleration metrics. Note: (**Left**) panels display the estimated probabilities of injury (Injured = Yes) as a function of acute:chronic workload ratios (ACWRs), with 95% confidence intervals (shaded area). (**Right**) panels show the ROC curves corresponding to each predictor, assessing the discriminative ability of the logistic regression models. The red lines represent the sensitivity–specificity trade-off across probability thresholds, with the area under the curve (AUC) quantifying model performance. (**A**) Week 3 (W3): Probability of injury and ROC curve for the ACWR of acceleration events recorded during the third week preceding injury occurrence. (**B**) Week 4 (W4): Probability of injury and ROC curve for the ACWR of acceleration events recorded during the fourth week preceding injury occurrence.

**Figure 4:**
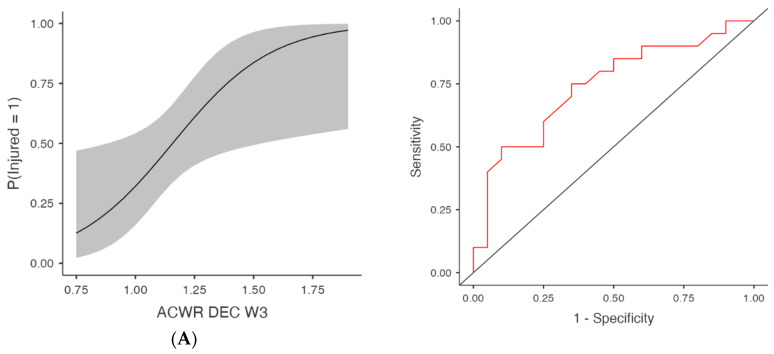

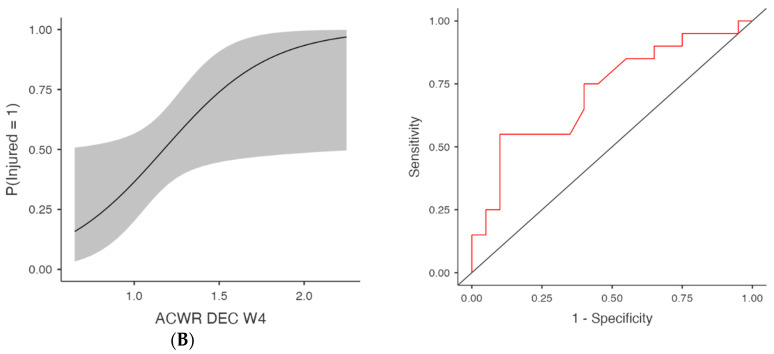
Injury Risk Probability and ROC Curves for Weekly ACWR- Deceleration metrics. Note: (**Left**) panels display the estimated probabilities of injury (Injured = Yes) as a function of acute:chronic workload ratios (ACWRs), with 95% confidence intervals (shaded area). (**Right**) panels show the ROC curves corresponding to each predictor, assessing the discriminative ability of the logistic regression models. The red lines represent the sensitivity–specificity trade-off across probability thresholds, with the area under the curve (AUC) quantifying model performance. (**A**) Week 3 (W3): Probability of injury and ROC curve for the ACWR of deceleration events recorded during the third week preceding injury occurrence. (**B**) Week 4 (W4): Probability of injury and ROC curve for the ACWR of deceleration events recorded during the fourth week preceding injury occurrence.

Initially, significant models were identified for multiple ACWR variables (Table 1 and Table 2, Figure 1, Figure 2, Figure 3 and Figure 4). ACWR DSR15–20 showed strong predictive power across all four weeks (Table 1 and Figure 1). At W3, the model yielded χ^2^(1) = 14.50, *p* < 0.001, R^2^McF = 0.262, R^2^N = 0.406 and an AUC of 0.811 (95% CI [0.675, 0.947]), indicating excellent discriminative ability.

The odds ratio for ACWR DSR15–20 W3 was 3104.00 (95% CI [12.60, 762,252.51], *p* = 0.004, FDR-adjusted *p* = 0.016). At W2, the same metric yielded χ^2^(1) = 11.00, *p* < 0.001, R^2^McF = 0.198, R^2^N = 0.319, with an AUC of 0.804 and OR = 1663.00 (95% CI [4.90, 564,248.35], *p* = 0.013, FDR-adjusted *p* = 0.019). Week 1 and Week 4 models for ACWR DSR15–20 were also significant. At Week 1, the model yielded χ^2^(1) = 8.29, *p* = 0.004, R^2^McF = 0.150, R^2^N = 0.250, with an AUC of 0.754 and OR = 57.49 (95% CI [2.02, 1633.77], *p* = 0.018, FDR-adjusted *p* = 0.019). At Week 4, the model yielded χ^2^(1) = 10.40, *p* = 0.001, R^2^McF = 0.188, R^2^N = 0.305, with an AUC of 0.791 and OR = 101.49 (95% CI [2.14, 4812.11], *p* = 0.019, FDR-adjusted *p* = 0.019).

Pairwise comparisons of AUC values were conducted using DeLong’s test (assuming correlation coefficient r = 0.5). Across-week comparisons revealed no significant differences (all *p* > 0.30), indicating stable discriminative capacity over time. Within-week contrasts showed a single significant result, where ACWR DSR15–20 demonstrated a higher AUC than ACWR TD during Week 2 (*Z* = −2.49, *p* = 0.01). All other comparisons were nonsignificant (*p* ≥ 0.05), confirming overall consistency across predictors and weeks.

ACWR DSR>25 was also significantly associated with injury occurrence (Table 1 and Figure 2). At W3, the model produced χ^2^(1) = 8.64, *p* = 0.003, AUC = 0.735 and OR = 9.55 (95% CI [1.69, 53.92], *p* = 0.011, FDR-adjusted *p* = 0.024). At W2, χ^2^(1) = 6.66, *p* = 0.010, AUC = 0.709, OR = 8.88 (95% CI [1.42, 55.68], *p* = 0.020, FDR-adjusted *p* = 0.024) and at W1, χ^2^(1) = 6.09, *p* = 0.014, AUC = 0.706, OR = 15.08 (95% CI [1.43, 159.28], *p* = 0.024, FDR-adjusted *p* = 0.024). Finally, ACWR DSR>25 W4 also reached significance with χ^2^(1) = 7.58, *p* = 0.006, AUC = 0.714 and OR = 6.37 (95% CI [1.39, 29.23], *p* = 0.017, FDR-adjusted *p* = 0.024).

Regarding the total distance, none of the ACWR TD models reached statistical significance. ACWR TD W3 showed a trend toward an association with injury risk (χ^2^(1) = 5.24, *p* = 0.022; R^2^McF = 0.094; AUC = 0.675; OR = 236.33, 95% CI [0.91, 61,385.02], *p* = 0.054), while ACWR TD W4 also demonstrated a marginal trend (*p* = 0.076; AUC = 0.665). However, both models failed to reach the conventional level of statistical significance.

Acceleration-based metrics (ACWR ACC) were also predictive in several models (Table 1 and Figure 3), at W3, χ^2^(1) = 8.05, *p* = 0.005, R^2^McF = 0.145, AUC = 0.737 and OR = 179.89 (95% CI [2.28, 14,196.67], *p* = 0.020) and at W4, χ^2^(1) = 7.61, *p* = 0.006, AUC = 0.755, OR = 55.91 (95% CI [1.61, 1937.43], *p* = 0.026). Week 1 showed trends towards significance with χ^2^(1) = 4.13, *p* = 0.042, AUC = 0.676 and OR = 162.02 (95% CI [0.82, 32,153.98], *p* = 0.059), as well as W2 (*p* = 0.075), although the AUC was 0.689. Deceleration metrics (ACWR DEC) were significantly associated with injury in multiple weeks (Table 1 and Figure 4), at W3, χ^2^(1) = 7.06, *p* = 0.008, R^2^McF = 0.127, AUC = 0.741 and OR = 117.63 (95% CI [1.63, 8480.99], *p* = 0.029) and at W4, χ^2^(1) = 5.71, *p* = 0.017, AUC = 0.720, OR = 24.48 (95% CI [1.10, 544.01], *p* = 0.043). ACWR DEC W1 and W2 showed trends toward significance but did not meet the threshold (*p* = 0.098 and *p* = 0.118, respectively), with AUC values of 0.655 and 0.682, respectively. However, the acceleration (ACC) and deceleration (DEC) models no longer reached significance after FDR adjustment. Specifically, although ACWR ACC W3 (FDR *p* = 0.052) and ACWR DEC W3 (FDR *p* = 0.086) initially indicated potential predictive trends (unadjusted *p* < 0.05), these effects were attenuated after controlling for multiple comparisons. Similarly, total distance (ACWR TD) and ACWR DSR20–25 did not reach significance in any week.

**Figure 5:**
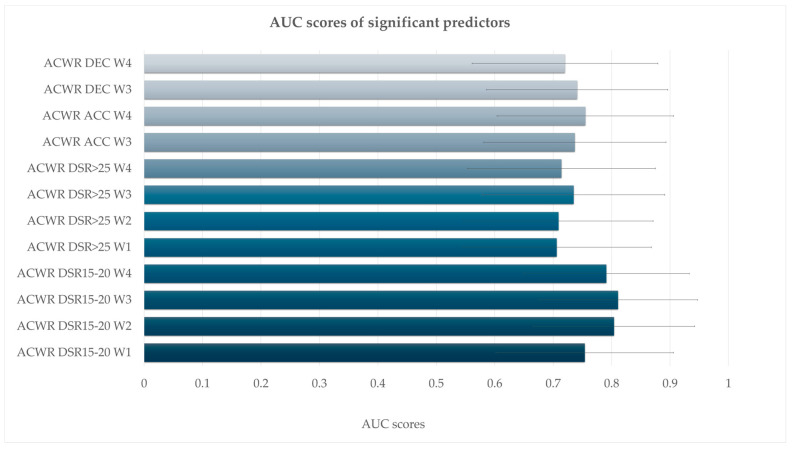
Area under the curve (AUC) values with 95% confidence intervals for acute:chronic workload ratio (ACWR) variables significantly associated with injury risk. All predictors listed demonstrated statistical significance (*p* < 0.05) in the logistic regression models.

In terms of model performance, most significant models demonstrated balanced classification metrics, with sensitivity and specificity values typically ranging from 0.65 to 0.80 (Table 2 and Figure 5). The models for ACWR DSR15–20 across W1–W4 had the highest classification accuracy (80%), supporting their utility in injury prediction. Estimated marginal means visualizations further illustrated the increased injury probability associated with higher ACWR values.

**Figure 6:**
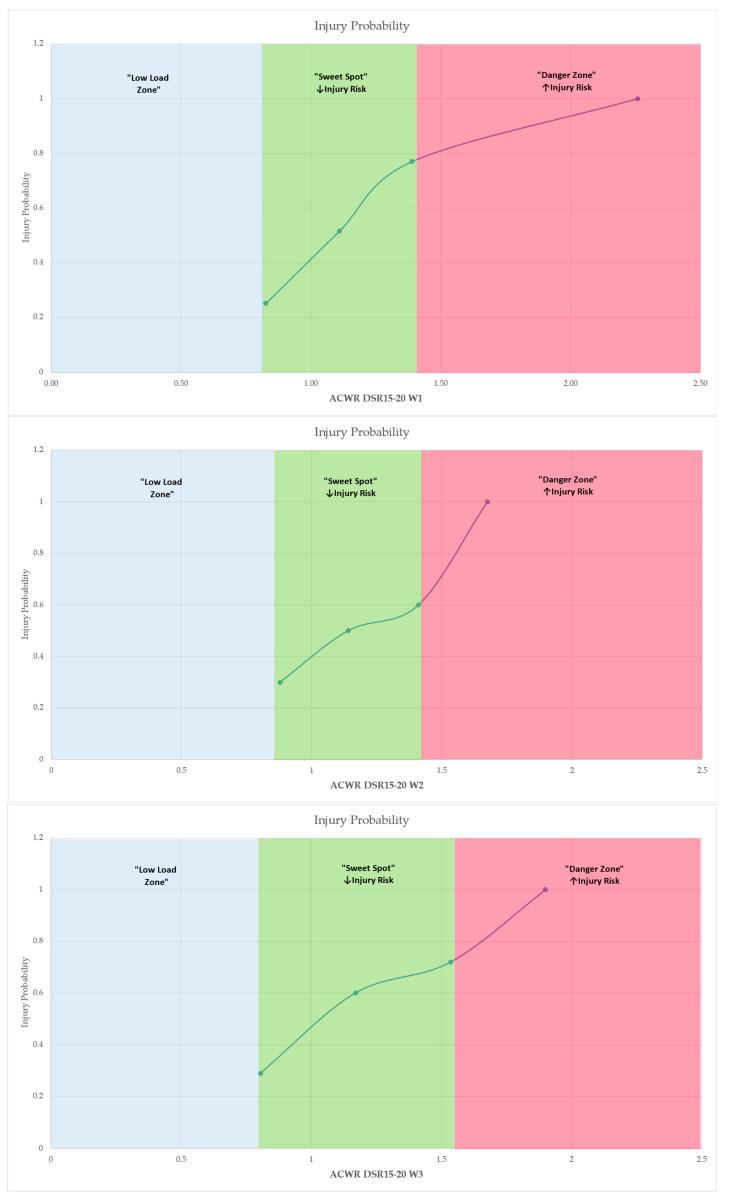

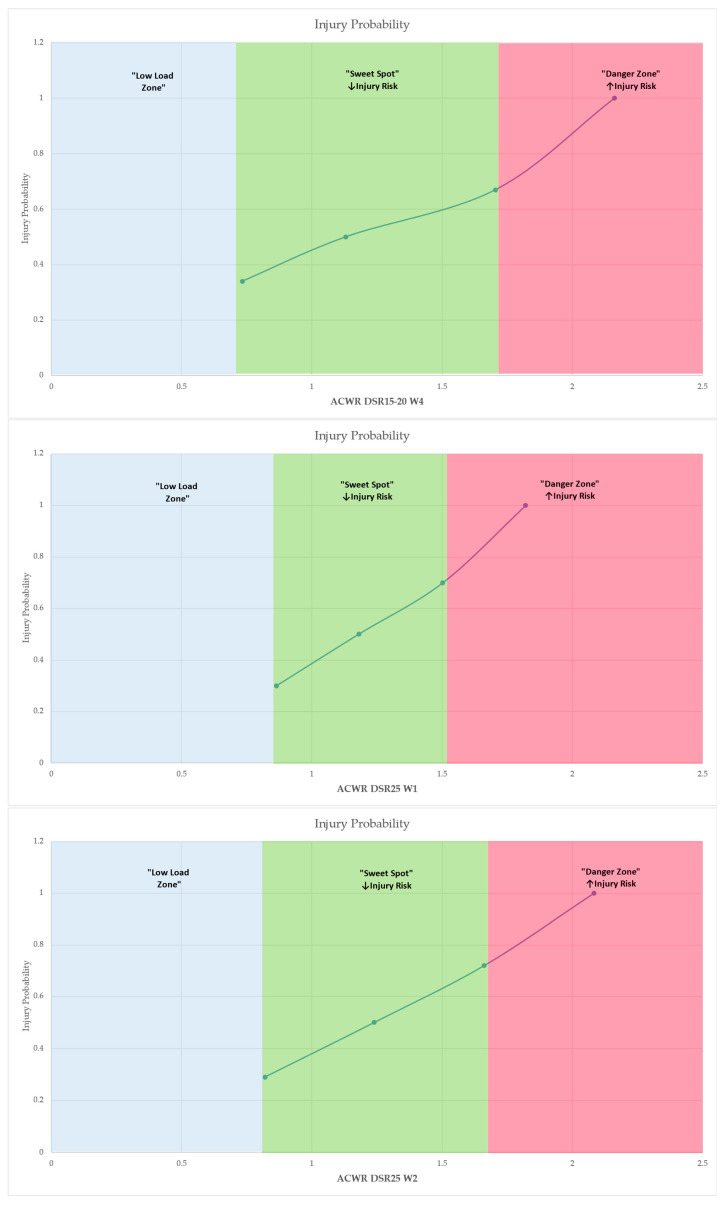

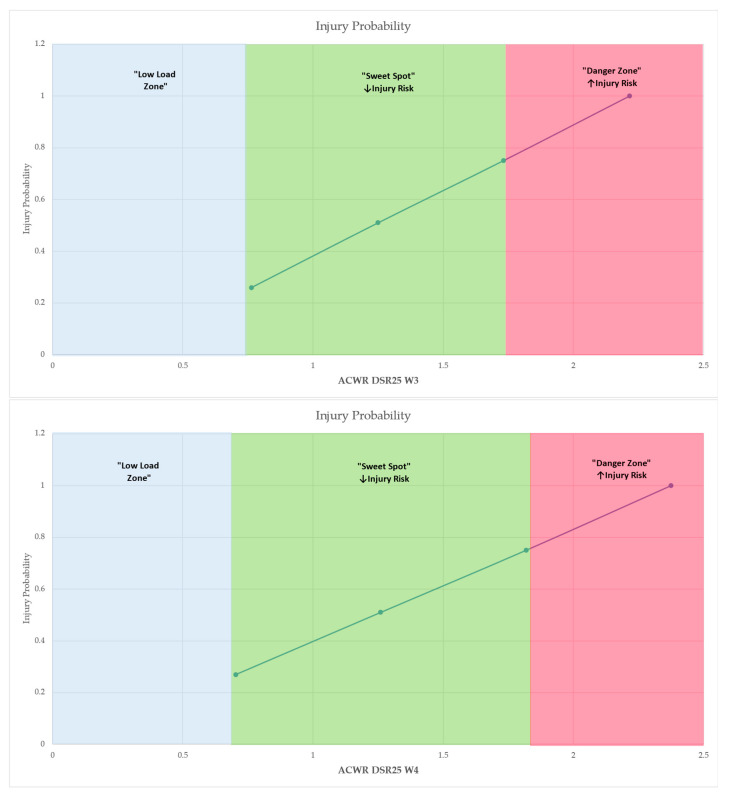
Probability of injury across ACWR values, illustrating the classification into “Low Load Zone,” “Sweet Spot” and “Danger Zone.” Shaded areas represent the respective risk zones derived from estimated marginal means. The “Low Load Zone” is denoted as exploratory, reflecting theoretical thresholds not empirically supported by the present in-season data. Arrows (↑, ↓) indicate an increase or decrease, respectively, in the probability of injury relative to changes in workload ratios. Only predictors that remained statistically significant after the false discovery rate (FDR) correction (q < 0.05) are presented.

To generate empirically based thresholds for injury risk prediction based on these results, ACWR values were classified into three zones, Low Load, Sweet Spot Danger Zone, based on binomial logistic regression models and estimation of marginal means across time points and workload categories (Table 3 and Figure 6).

The classification relied on the continuous predicted probabilities of injury generated by the regression models for each ACWR variable. Specifically, ACWR values at mean −1 standard deviation (−1 SD), mean and mean +1 standard deviation (+1 SD) were extracted through the EMMs function to visualize the probability rating of injury risk. The “sweet spot” zone was defined by the range of ACWR values where injury probability was moderate, corresponding closely to the sample mean. In contrast, values below −1 SD represented a low load zone, associated with low external stimulus but elevated injury probability due to suboptimal preparedness, while values above +1 SD constituted the danger zone, where overloading was statistically associated with significantly increased injury risk.

This zoning approach, based on estimated marginal means, delineated three distinct workload categories—Low Load, Sweet Spot, and Danger Zone—reflecting undertraining, optimal loading, and overload conditions, respectively. For instance, in the case of ACWR DSR15–20 W3, injury probability rose from 0.0737 at 0.805 (−1 SD) to 0.9656 at 1.535 (+1 SD), with the sweet spot anchored around 1.170 (mean), where probability remained around 0.59. Logistic regression analysis yielded highly significant associations (χ^2^ = 14.5, *p* < 0.001) and strong model fit metrics (AUC = 0.811; R^2^McF = 0.262), validating the discrimination power of this variable. Similar patterns were consistently observed for ACWR DSR15–20 across weeks 1–4 and for ACWR DSR>25, ACC and DEC metrics. Consequently, Table 3 and Figure 6 summarize the defined workload zones with the model-derived thresholds (Low Load, Sweet Spot, and Danger Zone) for each variable identified as a statistically significant predictor in the binomial logistic regression models across the four-week analysis period.

In addition to the primary univariate binomial logistic regressions (one ACWR predictor per model), parsimonious multivariable models were estimated to assess the robustness of the ACWR–injury associations. Each ACWR variable was entered individually and adjusted for age (years) and playing position (DEF as the reference; indicators for MID and ATT). Likelihood-ratio tests, Wald coefficients with odds ratios (ORs) and 95% confidence intervals (CIs), as well as AUC values with 95% CIs, were computed for each adjusted model. In the adjusted analyses, the pattern of results closely paralleled the univariate findings. Age and playing position were not associated with injury risk in any model (all *p* ≥ 0.15), and the ACWR effects remained stable. ACWR DSR15–20 persisted as a consistent predictor across W1–W4 (likelihood-ratio tests: W1 χ^2^ = 9.21, *p* = 0.002; W2 χ^2^ = 11.23, *p* < 0.001; W3 χ^2^ = 14.11, *p* < 0.001; W4 χ^2^ = 9.21, *p* = 0.002), with AUCs ranging from 0.79 to 0.84. ACWR DSR>25 also remained significant across W1–W4 (W1 *p* = 0.015; W2 *p* = 0.022; W3 *p* = 0.008; W4 *p* = 0.014; AUCs ≈ 0.73–0.76). ACWR ACC showed significant associations in W3–W4 (W3 *p* = 0.003, AUC = 0.785; W4 *p* = 0.008, AUC = 0.773) and marginal trends in W1–W2. ACWR DEC was significant in W3–W4 (W3 *p* = 0.006, AUC = 0.787; W4 *p* = 0.025, AUC = 0.755). ACWR DSR20–25 remained nonsignificant across all weeks, while ACWR TD exhibited only a marginal trend (e.g., W3 likelihood-ratio *p* = 0.034; AUC ≈ 0.715) with wide confidence intervals.

## 4. Discussion

The present study examined the predictive value of ACWR for non-contact injury risk in professional soccer players, utilizing binomial logistic regression models with week-by-week stratification across the four weeks preceding injury, in order to advance the current state of research by providing probabilistic injury risk estimations and identifying workload zones (“Low Load,” “Sweet Spot,” and “Danger Zone”) for external load indicators. The study’s findings offer significant practical implications for load management and injury prevention, building on but also challenging prior normative thresholds, such as the 0.80–1.30 ACWR range proposed by Gabbett et al. [19].

When comparing the current results to Gabbett’s ACWR “sweet spot” of 0.80–1.30, it should be considered that contemporary research conducted by Bowen and colleagues concluded that both high- (ACWR = 1.24) and low-risk (ACWHR = 1.13) groups experienced an increased likelihood of injury given their distinct types of stress [6]. Moreover, in 2020, a significantly higher injury risk was observed in English Premier League players when ACWR surpassed 2.0, particularly for HSR loads. The higher tiers found in our study might be due to some reasons. The first is that soccer temporally demands (e.g., congestion and microcycling) may provide higher acute peaks for players while preserving a good chronic condition, resulting in push-ups of the risk curve. Secondly, the comparison of methodological discrepancies in ACWR calculation wherein rolling averages rather than an exponentially weighted moving average (EWMA) may attenuate the appearance of short-term fluctuations resulting in a lower apparent sensitivity to load spikes. Furthermore, aspects related to each population (league level, training culture and medical supervision intensity) also may influence the manner by which load ratios are translated into injury risk. Finally, it should be emphasized that the ACWR zones reported here (mean ± 1 SD) are sample-specific descriptive ranges derived from the current dataset. They should not be interpreted as universal “optimal load zones” or applied clinically without external validation in independent cohorts and competitive contexts.

The analysis of the ACWR variables revealed that moderate-intensity running loads, as represented by ACWR DSR15–20 (distance covered at 15–20 km/h), consistently emerged as the most significant predictor of non-contact injury risk across all four weeks prior to injury occurrence. Particularly, week 3 demonstrated the highest discriminative capacity (AUC = 0.811), indicating excellent model performance. Week 3 demonstrates significant correlations in an injury and may represent a pivotal timeframe in which cumulative tissue microtrauma meets or exceeds the threshold. It seems that neuromuscular fatigue accumulation, across the 2–3 week time frame, can cause protective mechanisms’ injury impairment.

Across the other weeks, ACWR DSR15–20 maintained high levels of classification accuracy, sensitivity, and specificity (80% across weeks 2–4), supporting its utility as a primary workload indicator for injury prevention strategies. In line with these findings, the zoning analysis established clear thresholds, with a ‘sweet spot’ zone of 0.805–1.535 for DSR15–20 at week 3, and values exceeding 1.535 falling within the ‘danger zone.’ This suggests that even within the same workload metric, careful attention must be paid to week-specific variations, as exceeding these thresholds, resulting from the current study, significantly elevated injury risk. This finding partially overlaps with Gabbett’s [19] proposed range but suggests that the upper limit for moderate running intensity may be higher in this specific soccer population than previously assumed. Moreover, it enhances the growing recognition of moderate-speed running as a central factor in injury development and aligns with the work of previous studies [9,10], which have argued that while high-intensity efforts are important for match preparation, cumulative stress in moderate-intensity zones can lead athletes to overuse injuries if not properly managed.

Specifically, Gabbett’s framework [19] suggests that maintaining an ACWR between 0.80 and 1.30 minimizes injury risk, with values below 0.80 indicating undertraining and values above 1.30 associated with overload and high injury risk. However, these thresholds were developed across multiple sports contexts and not specifically validated through multivariate predictive modeling or soccer-specific data. In contrast, the present study employed logistic regression models with ROC analysis to derive injury risk probabilities, offering a data-driven stratification based on actual injury occurrences within an elite soccer team. When comparing our results with these thresholds [19], which suggests that ACWR values below 0.80 or above 1.30 significantly increase injury risk, a notable distinction is indicated. The sweet spot zones in our study for DSR15–20 extended beyond the traditional upper boundary, with danger zones being defined above 1.535 at week 3. Regarding the ACWR threshold, one study has suggested a significantly higher value [19], emphasizing the importance of the model, while also suggesting that the injury risk threshold may be above 2.0. This variation may be attributed to the specific context of elite soccer, where repetitive efforts in the moderate-speed range are particularly demanding, especially for positions such as midfielders known to sustain higher locomotor loads [32]. Furthermore, it is noteworthy that, although midfielders were found to be at highest overall injury risk, our sample size did not allow for position-stratified regression analyses with adequate power. Thus, although the positional role was controlled for in all models as a covariate and the moderation effect of position could only be weakly tested. This should be recognized and future research should try to derive position-specific ACWR cut-off points representing the specific locomotor and metabolic demands of various playing positions. Therefore, the stiff application of a universal 0.80–1.30 window may not sufficiently capture the nuanced workload demands experienced by different playing roles and intensity zones. Additionally, it is important to highlight that in the one to two weeks leading up to an injury, a lower ACWR threshold might be necessary. Even when reduced, the threshold continues to play a key role in injury prevention, as shown in our earlier research [25]. Future research employing larger, multi-team datasets should seek to establish externally validated cut-points, report their sensitivity and specificity, and consider alternative criteria, such as cost-sensitive thresholds or decision-curve analyses, to enhance the applied and clinical utility of ACWR-based injury prediction models.

Similarly, high-speed running (ACWR DSR > 25, distance covered at >25 km/h) also showed consistent predictive capacity, particularly at weeks 3 (AUC = 0.735) and 4 (AUC = 0.714), where our defined sweet spot extended up to approximately 1.73 in week 3, again going beyond the traditional 1.30 threshold suggested by Gabbett et al. [19]. This indicated that sprinting activities, critical for match performance, may require greater flexibility in load management strategies. Such findings align with previous studies emphasizing the necessity of exposing athletes to high-speed efforts to foster tissue resilience and match-specific preparedness [9,10,11]. In every case, injured athletes exhibited higher mean values for high intensities compared to non-injured athletes, aligning with previous findings [39,40]. Moreover, it has been demonstrated that increased training and match intensity contributes to injury occurrence [41]. The strict adherence to a universal 1.30 upper limit may therefore risk underpreparation in sprint-related performance capacities, elevating potential injury risk when match demands exceed training exposures. Insufficient load exposure compromises physiological preparedness as described by Blanch and Gabbett [19]. Particularly in elite soccer, where match play involves unpredictable high-intensity efforts, insufficient load accumulation may leave athletes unprepared to cope with the demands of the game.

Regarding total distance (ACWR TD), it narrowly did not reached significance threshold (AUC = 0.675), suggesting a more limited but still relevant contribution of total distance load in injury prediction. The relatively lower AUC and explained variance compared to DSR15–20 and DSR>25 may reflect the non-specificity of total distance, which captures overall volume but not the qualitative aspects of high-intensity efforts, accelerations, or decelerations that implement greater mechanical strain. Additionally, the moderate predictive values observed for ACWR DSR20–25 (AUC up to 0.702 at week 3) highlight its supplementary role, although it did not consistently reach the same level of predictive accuracy as DSR15–20 or DSR>25.

The contribution of this study is the week-by-week analysis, which revealed that the third and fourth weeks prior to injury represent the periods of highest predictive value for several ACWR metrics. Simultaneously, our results are supported by studies [42,43] that examined injury likelihood using the ACWR over the preceding four weeks, focusing on metrics like high-intensity speed, acceleration and deceleration. These studies reported a higher probability of injury when thresholds were generally exceeded (>1.30) compared to the moderate ACWR range. Earlier research emphasized also the final week before injury for its significant decrease in various performance indicators [23]. This could suggest that critical maladaptation processes may initiate well before the immediate pre-injury week, supporting the need for longitudinal monitoring and early intervention strategies. Particularly, the strong predictive performance of DSR15–20 and DSR>25 at W3 implies that training load adjustments implemented only in the final week may be insufficient to moderate injury risk.

Acceleration (ACC) and deceleration (DEC) metrics further confirmed this pattern, with significant models observed specifically in W3 and W4, highlighting that the third and fourth weeks prior to injury are critical periods where workload monitoring should be particularly systematically gathered [23]. A potential link was also identified between non-contact injuries and accelerations, and to a lesser extent, decelerations. This aligns with previous research [44], which highlighted the significant role of concentric and eccentric movements in certain injuries. Furthermore, a study on young elite-level soccer players [45] found that accelerations over a three-week period were the strongest predictor of injury. ACWR ACC in week 3 demonstrated excellent predictive capacity (AUC = 0.737), while in week 4, similar predictive value was identified (AUC = 0.755). In parallel, deceleration load (ACWR DEC) was significantly associated with injury occurrence at week 3 (AUC = 0.741) and week 4 (AUC = 0.720). These findings emphasize the necessity of detailed and systematic workload data collection, monitoring and management during these high-risk periods, particularly for acceleration and deceleration stimuli, where injury risk escalates beyond the identified ‘sweet spot’ zones (ACC W3: 0.932–1.395; DEC W3: 0.944–1.394) into the ‘danger zone’ when these thresholds are exceeded. These rapid changes in movement demand essential eccentric control and are known contributors to soft tissue strain [3,4,13]. The above chronological dimension provided valuable details to the existing literature, which frequently approaches workload ratios in a cumulative, summarized or averaged manner, rather than accounting for week-by-week fluctuations and their potential influence on injury risk [24]. Subsequently, the predictive utility of high-speed running (DSR>25), ACC, and DEC were also confirmed, especially during weeks 3 and 4, supporting earlier findings that these high-intensity actions contribute to tissue fatigue and microtrauma [3,6].

The observed deviations between our data-driven thresholds and the classical ACWR norms suggest that applying fixed, sport-generic zones may oversimplify the complex interplay between load, adaptation, and injury risk. Some models produced wide confidence intervals and inflated odds ratios, indicating potential quasi-separation and small-event bias. Extremely high odds ratios (e.g., OR > 1000 in Table 1) may represent artifacts of sparse data or quasi-separation within the logistic models, reflecting the limited number of injury events rather than true effect size. No internal validation procedures (e.g., k-fold cross-validation, split-sample, or bootstrapping) or formal calibration assessments were conducted; therefore, the generalizability and calibration accuracy of these models remain uncertain. Future studies should incorporate internal validation, report calibration metrics and plots, and compare results with simpler baseline models to determine whether parsimony provides greater stability with comparable discriminative capacity. While the 0.80–1.30 range provides a valuable starting point, our results underscore the necessity for population-specific and metric-specific calibration. Moreover, the use of logistic regression modeling allowed for continuous probability estimates, enabling the identification of optimal ranges grounded in actual injury data. These findings support a shift toward individualized load monitoring, where sweet spot and danger zone definitions are tailored to the specific demands of the sport, playing position, and individual athlete history. Our research focused on this aspect to enable a fair comparison between injured and non-injured players in the same positions, naturally considering the unique demands associated with each role on the field. Given that performance differs by position during a match [46], individualized training is essential within each microcycle. Furthermore, the integration of these probabilistic models into real-time monitoring systems could facilitate early warnings when athletes approach high-risk zones, enhancing the responsiveness of injury prevention strategies.

While this study offers a novel contribution by applying binomial logistic regression and week-by-week stratification to estimate injury probability from external load indicators, the interpretation of the “low load” zone within the ACWR model requires caution. Although higher ACWR values were significantly associated with increased injury risk, the model may not fully capture risks related to undertraining. Given the in-season context, where workloads are relatively stable, opportunities to observe the effects of low ACWR values were limited. Although the present study delineated a “Low-Load Zone,” this classification must be interpreted with caution. The in-season dataset contained few very low-load exposures; therefore, these thresholds are exploratory and included mainly for visualization and theoretical completeness. They should be viewed as hypothesis-generating, pending confirmation in studies that deliberately sample under-loading phases (e.g., pre-season or return-to-play) and allow for non-linear modeling of load–injury relationships. Future studies incorporating a larger number of injury events should also estimate ROC-derived cut-offs (e.g., Youden index) to identify optimal decision thresholds and report their sensitivity, specificity, and external validation across independent datasets. Additionally, comparing alternative criteria such as cost-sensitive thresholds or decision-curve analysis could enhance the applied usefulness and clinical interpretability of ACWR-based injury risk models. Future studies also could examine the full aspects of workload dynamics by incorporating larger, multi-team datasets and applying more flexible statistical methodologies, such as generalized additive models (GAMs) or machine learning techniques, to explore potential non-linear associations. To address small-event bias and improve robustness, future investigations should consider penalized logistic regression (e.g., Firth correction, ridge), bootstrap-based validation, and regularized machine-learning methods to capture potential non-linear associations while controlling overfitting and maintaining interpretability. Such approaches may provide deeper insights into whether both undertraining and overtraining contribute to heightened injury risk. Addressing these aspects would further strengthen injury prevention strategies and support more precise, individualized load management protocols—particularly in the demanding environment of elite soccer, where optimizing performance while minimizing injury risk remains a fundamental challenge.

Moreover, the interpretation of the Low Load Zone should be approached with caution. The small number of observations within this range limits the statistical power to determine precise thresholds or confidently model the associated injury risk. Consequently, the values reported for the Low Load Zone are considered exploratory and intended to illustrate potential trends rather than definitive cut-off points. Future investigations with larger and more balanced datasets are required to clarify whether very low training loads truly confer a protective effect or if other contextual factors, such as recovery status and individual variability, moderate this relationship.

Furthermore, the presence of wide confidence intervals and inflated odds ratios in some models likely reflects the limited number of injury observations and the resulting statistical instability. Although the present analysis did not include internal validation procedures such as cross-validation or bootstrapping, these techniques are recommended for future research to evaluate model calibration and generalizability. Accordingly, the observed associations should be viewed as indicative rather than conclusive, reflecting potential patterns that warrant further investigation. Subsequent studies incorporating larger and more diverse samples, penalized regression approaches), and model validation techniques are necessary to confirm the robustness, transferability, and predictive reliability of these findings across different competitive contexts.

In addition to the methodological constraints, some further limitations should be acknowledged. First, the relatively small sample size may limit the generalizability of the findings, and the conclusions may not extrapolate to female athletes, youth populations, or other sports disciplines. Second, the study’s design does not allow causal inferences to be drawn; confounding factors such as individual recovery practices, previous injury history, or playing surface conditions could have influenced injury risk. Third, contextual variables, including match congestion, psychological stress, and varying match intensity, were not systematically controlled or analyzed, potentially affecting workload–injury relationships. Fourth, potential multicollinearity among ACWR indicators should be considered, since workload variables such as total distance, running distance, and acceleration counts are often correlated, which might have influenced the independent predictive power of each variable. Fifth, several models produced inflated odds ratios accompanied by wide confidence intervals, likely reflecting the limited number of injury events (0.4 per week on average) and potential quasi-separation effects within the logistic regression analyses. Accordingly, these estimates should be interpreted as indicative of the direction and relative strength of association rather than as precise effect magnitudes. Future research employing larger datasets or more advanced statistical approaches is warranted to verify the stability and robustness of these associations. Sixth, although a false discovery rate (FDR) correction was applied to mitigate Type I error inflation arising from multiple comparisons, the possibility of residual false-positive results cannot be completely excluded. Therefore, the findings should be interpreted with appropriate caution. Finally, the study was conducted within the framework of existing regulations and mandatory rest periods governing elite player workload management, which may have further constrained variability in the dataset.

### Research/Policy Implications

The findings highlight the need for tailored injury prevention protocols that consider player-specific risk factors, suggesting that team medical staff and policymakers in professional soccer leagues should integrate individualized monitoring systems into regular training routines. Given the long-term trends observed in injury incidence, there is a clear rationale for federations to establish standardized data collection frameworks across clubs to facilitate early detection of high-risk profiles and enable more effective policy interventions at the league level. Future research should focus on the impact of evolving game demands, such as increased match intensity and fixture congestion, to inform evidence-based regulations regarding player workload management and mandatory rest periods. Practically, the present results offer actionable information to monitor load and avoid structural damage in professional football. When ACWR values exceed certain thresholds (mainly in high-speed running and sprinting distances), coaches and sports scientists could act by modifying the structure of the microcycle, changing subsequent exposure to high velocity actions or including low-load recovery sessions while prioritizing more often technical-tactical tasks over a new day with fitness aimed work for about 24 h. In addition, including real-time monitoring systems (e.g., GPS-based dashboards or automated alerts) can facilitate the identification of load peaks soon after they occur, despite current barriers being data validity, staff capability and resource constraints that could delay their implementation. Professionals must also comprehend that there is an inherent dichotomy between maximizing readiness and reducing injury risk, with some provincial overload probably required from time to time to induce training responses (you cannot make progress without stressing the systems). Thus, personalized monitoring—considering the history, position and match demands of the players—is still needed. Policy future developments should promote the standardization of ACWR monitoring protocols and development of position- and context-specific thresholds capable to enable safer, evidence-based management, throughout professional leagues.

## 5. Conclusions

The analysis demonstrated that ACWR DSR15–20 was consistently indicated as the strongest predictor of injury across all four weeks, with the highest discriminative power observed during week 3. In addition, high-speed running load (ACWR DSR>25), acceleration (ACWR ACC), and deceleration (ACWR DEC) metrics were identified as significant predictors in several models, particularly during weeks 3 and 4 preceding injury occurrence. These findings emphasize the critical relationship between high and moderate-intensity running loads and injury risk, highlighting the importance of carefully monitoring and managing exposure within this speed range to enhance injury prevention strategies in elite soccer players. In addition, the categorization of workload into “Low Load,” “Sweet Spot,” and “Danger Zone” based on the estimated marginal means derived from the current study’s data provided thresholds to inform injury prevention strategies, practical guidance for load monitoring, and support evidence-based decision-making in elite soccer contexts. Specifically, for ACWR DSR15–20 (moderate-speed running), the “Sweet Spot” narrowed progressively across the four weeks leading up to injury, indicating increasing sensitivity to load deviations as injury risk approached. At W1, the Sweet Spot ranged from 0.960 to 1.480, while values below 0.960 indicated undertraining (Low Load), and values above 1.480 suggested overload (Danger Zone). At W2, the optimal range narrowed to 0.879–1.408. By W3, the optimal range narrowed to 0.805–1.535, with higher injury probability observed outside this interval. At W4, the thresholds shifted further (Sweet Spot: 0.733–1.705), reflecting variability in adaptive load tolerance over time. For ACWR DSR>25, a similar progression was evident. At W1, the Sweet Spot was 0.865–1.503; the Danger Zone began above 1.503. By W4, this threshold increased, with overload defined as ACWR > 1.818 and undertraining as <0.704, suggesting greater risk sensitivity to sprinting load in the immediate pre-injury period. Acceleration-based loads (ACWR ACC) at W3 showed an optimal range between 0.932 and 1.395. Values below 0.932 or above 1.395 were associated with significantly elevated injury risk. At W4, these thresholds shifted slightly to 0.901–1.471, maintaining a similar pattern of load sensitivity.

Deceleration metrics (ACWR DEC) followed a comparable trend. At W3, the Sweet Spot spanned 0.944–1.394, while at W4, it ranged from 0.896 to 1.481. Exceeding the upper bounds of these ranges was consistently associated with a marked rise in injury probability. Coaching staff may utilize the identified “sweet spot” zones to guide weekly load adjustments, aiming to maintain athletes within optimal ranges that minimize injury risk while still promoting sufficient physical adaptation. Conversely, the detection of “danger zone” values may prompt preventive load reduction, individualized recovery protocols, or alternative training prescriptions for at-risk athletes, particularly in the two-to-four weeks preceding competitive or important matches. By adopting a probabilistic injury risk model, these findings could contribute to the development of evidence-based load management strategies aimed at reducing injury incidence, optimizing training periodization, and supporting long-term athlete health and performance. These results may serve as a foundation for further research and intervention development in the field of sports science and injury prevention, potentially enhanced through the implementation of artificial intelligence (AI)-based models, including machine learning algorithms and predictive analytics. Such approaches could further improve the precision of injury risk estimation by integrating multivariate workload indicators, individual athlete profiles, and contextual factors, thereby supporting more accurate and personalized load management strategies. The use of the limits presented in the study with specific reference values will be able to enhance their usefulness for injury prevention practices as well as for monitoring training loads with regard to maximizing athletic performance. The study encourages the use of these results between different leagues or age groups, which will probably contribute to the validation of the specific limits in order to support their application in practice, through scientific documentation.

## Figures and Tables

**Table 1:** Binary Logistic Regression Predicting Injury Likelihood.

Predictor	R^2^McF	R^2^N	B (Estimate)	SE	Odds Ratio (OR)	95% CI [Lower, Upper]	*p*	FDR- Adjusted *p*
ACWR TD W1	0.030	0.054	3.71	2.97	40.87	[0.12–13,674.51]	0.211	0.152
ACWR TD W2	0.030	0.055	2.89	2.27	17.96	[0.21–1551.24]	0.204	0.152
ACWR TD W3	0.094	0.164	5.47	2.84	236.33	[0.91–61,385.02]	0.054	0.211
ACWR TD W4	0.068	0.119	3.71	2.09	40.70	[0.68–2442.57]	0.076	0.211
ACWR DSR15-20 W1	0.150	0.250	4.05	1.71	57.49	[2.02–1633.77]	0.018 *	0.019 ‡
ACWR DSR15-20 W2	0.198	0.319	7.42	2.97	1663.00	[4.90–564,248.35]	0.013 *	0.019 ‡
ACWR DSR15-20 W3	0.262	0.406	8.04	2.81	3104.00	[12.60–762,252.51]	0.004 *	0.016 ‡
ACWR DSR15-20 W4	0.188	0.305	4.62	1.97	101.49	[2.14–4812.11]	0.019 *	0.019 ‡
ACWR DSR20-25 W1	0.045	0.081	2.76	1.83	15.85	[0.44–577.21]	0.132	0.140
ACWR DSR20-25 W2	0.043	0.078	2.04	1.38	7.66	[0.51–114.70]	0.140	0.140
ACWR DSR20-25 W3	0.059	0.104	2.24	1.33	9.39	[0.70–126.50]	0.091	0.140
ACWR DSR20-25 W4	0.078	0.136	2.24	1.18	9.38	[0.94–94.07]	0.057	0.140
ACWR DSR>25 W1	0.110	0.188	2.71	1.20	15.08	[1.43–159.28]	0.024 *	0.024 ‡
ACWR DSR>25 W2	0.120	0.205	2.18	0.94	8.88	[1.42–55.68]	0.020 *	0.024 ‡
ACWR DSR>25 W3	0.156	0.259	2.26	0.88	9.55	[1.69–53.92]	0.011 *	0.024 ‡
ACWR DSR>25 W4	0.137	0.230	1.85	0.78	6.37	[1.39–29.23]	0.017 *	0.024 ‡
ACWR ACC W1	0.075	0.131	5.09	2.70	162.02	[0.82–32,153.98]	0.059	0.075
ACWR ACC W2	0.066	0.116	3.67	2.06	39.22	[0.69–2227.41]	0.075	0.075
ACWR ACC W3	0.145	0.243	5.19	2.23	179.89	[2.28–14,196.67]	0.020 *	0.052
ACWR ACC W4	0.137	0.230	4.02	1.81	55.91	[1.61–1937.43]	0.026 *	0.052
ACWR DEC W1	0.050	0.088	4.36	2.79	78.63	[0.33–18,754.49]	0.118	0.118
ACWR DEC W2	0.057	0.100	3.34	2.00	28.28	[0.56–1414.25]	0.094	0.118
ACWR DEC W3	0.127	0.216	4.77	2.18	117.63	[1.63–8480.99]	0.029 *	0.086
ACWR DEC W4	0.103	0.177	3.20	1.58	24.48	[1.10–544.01]	0.043 *	0.086

* *p* < 0.05, ‡ q < 0.05. Note: Asterisks (*) indicate unadjusted significance; double daggers (‡) indicate significance after FDR adjustment (q < 0.05).

**Table 2:** Predictive Performance Metrics (Accuracy, Sensitivity, Specificity and AUC) of Binomial Logistic Regression Models by ACWR Variable and Week.

Predictor	AUC	95% CI [Lower, Upper]	Accuracy	Sensitivity	Specificity	PPV	NPV
ACWR TD W1	0.625	[0.451, 0.799]	0.600	0.550	0.650	0.61	0.59
ACWR TD W2	0.600	[0.423, 0.777]	0.575	0.600	0.550	0.57	0.58
ACWR TD W3	0.675	[0.507, 0.843]	0.650	0.600	0.700	0.67	0.64
ACWR TD W4	0.665	[0.496, 0.834]	0.650	0.650	0.650	0.65	0.65
ACWR DSR15-20 W1	0.754	[0.602, 0.906]	0.725	0.700	0.750	0.74	0.71
ACWR DSR15-20 W2	0.804	[0.666, 0.942]	0.750	0.750	0.750	0.75	0.75
ACWR DSR15-20 W3	0.811	[0.675, 0.947]	0.800	0.800	0.800	0.80	0.80
ACWR DSR15-20 W4	0.791	[0.649, 0.933]	0.800	0.800	0.800	0.80	0.80
ACWR DSR20-25 W1	0.645	[0.473, 0.817]	0.600	0.550	0.650	0.61	0.59
ACWR DSR20-25 W2	0.665	[0.496, 0.834]	0.575	0.600	0.550	0.57	0.58
ACWR DSR20-25 W3	0.702	[0.539, 0.865]	0.725	0.650	0.800	0.76	0.69
ACWR DSR20-25 W4	0.689	[0.524, 0.854]	0.725	0.700	0.750	0.74	0.71
ACWR DSR>25 W1	0.706	[0.544, 0.868]	0.625	0.650	0.600	0.62	0.63
ACWR DSR>25 W2	0.709	[0.547, 0.871]	0.625	0.600	0.650	0.63	0.62
ACWR DSR>25 W3	0.735	[0.579, 0.891]	0.675	0.650	0.700	0.68	0.67
ACWR DSR>25 W4	0.714	[0.553, 0.875]	0.650	0.550	0.750	0.69	0.62
ACWR ACC W1	0.676	[0.509, 0.843]	0.675	0.650	0.700	0.68	0.67
ACWR ACC W2	0.689	[0.524, 0.854]	0.575	0.550	0.600	0.58	0.57
ACWR ACC W3	0.737	[0.581, 0.893]	0.675	0.600	0.750	0.71	0.65
ACWR ACC W4	0.755	[0.604, 0.906]	0.700	0.650	0.750	0.72	0.68
ACWR DEC W1	0.655	[0.485, 0.825]	0.600	0.600	0.600	0.60	0.60
ACWR DEC W2	0.682	[0.516, 0.848]	0.550	0.550	0.550	0.55	0.55
ACWR DEC W3	0.741	[0.586, 0.896]	0.675	0.700	0.650	0.67	0.69
ACWR DEC W4	0.720	[0.561, 0.879]	0.600	0.550	0.650	0.61	0.59

Note: AUC = Area Under the Curve; PPV = Positive Predictive Value; NPV = Negative Predictive Value; ACWR = Acute: Chronic Workload Ratio; TD = Total Distance; DSR15–20 = Distance covered while running at >15–20 km/h; DSR20–25 = Distance covered while running at >20–25 km/h; DSR > 25 = Distance covered while sprinting at >25 km/h; ACC = Accelerations > 2.5 m/s^2^; DEC = Decelerations > 2.5 m/s^2^. PPV and NPV were computed from sensitivity and specificity using the formulas PPV = Se/(Se + 1 − Sp) and NPV = Sp/(Sp + 1 − Se), assuming an injury prevalence of 50% (20 injured, 20 non-injured players) and a classification threshold of 0.50. These indices represent the probability that a positive or negative model prediction corresponds to a true injury or non-injury, respectively.

**Table 3:** ACWR Zones for Injury Risk Classification.

ACWR Variable	Low Load Zone	Sweet Spot Zone	Danger Zone
ACWR DSR15-20 W1	<0.960	0.960–1.480	>1.480
ACWR DSR15-20 W2	<0.879	0.879–1.408	>1.408
ACWR DSR15-20 W3	<0.805	0.805–1.535	>1.535
ACWR DSR15-20 W4	<0.733	0.733–1.705	>1.705
ACWR DSR>25 W1	<0.865	0.865–1.503	>1.503
ACWR DSR>25 W2	<0.820	0.820–1.662	>1.662
ACWR DSR>25 W3	<0.764	0.764–1.731	>1.731
ACWR DSR>25 W4	<0.704	0.704–1.818	>1.818
ACWR ACC W3	<0.932	0.932–1.395	>1.395
ACWR ACC W4	<0.901	0.901–1.471	>1.471
ACWR DEC W3	<0.944	0.944–1.394	>1.394
ACWR DEC W4	<0.896	0.896–1.481	>1.481

## Data Availability

The availability of the data is open access for all interested parties if they contact the corresponding author via email.
